# Analysis of the Thermal Insulation and Fire-Resistance Capacity of Particleboards Made from Vine (*Vitis vinifera* L.) Prunings

**DOI:** 10.3390/polym12051147

**Published:** 2020-05-17

**Authors:** Manuel Ferrandez-Villena, Clara Eugenia Ferrandez-Garcia, Teresa Garcia-Ortuño, Antonio Ferrandez-Garcia, Maria Teresa Ferrandez-Garcia

**Affiliations:** Department of Engineering, Universidad Miguel Hernandez, 03300 Orihuela, Spain; cferrandez@umh.es (C.E.F.-G.); tgarcia@umh.es (T.G.-O.); antonio.ferrandezg@umh.es (A.F.-G.); mt.ferrandez@umh.es (M.T.F.-G.)

**Keywords:** plant waste, physical, mechanical, thermal and fire-resistance properties

## Abstract

In Europe, vine (*Vitis vinifera* L.) prunings are one of the most abundant types of agricultural waste. It is, therefore, essential to organize the removal of vine waste from the fields in order to prevent the spread of fires, pests, or diseases. Using plant biomass in buildings will help achieve greater energy efficiency and cause less environmental pollution. The objectives of this work were to minimize burning of agricultural waste, reduce the use of natural wood, and obtain a product by using vine pruning waste to manufacture particleboards, assessing their use as an insulating material and their fire-resistance qualities. Eight types of boards were manufactured with vine prunings (two particle sizes, two times, and two pressures), using 9% by weight of urea-formaldehyde as a bonding resin. Experimental tests were conducted to determine the physical, mechanical, thermal, and fire-resistance properties. In general, the panels manufactured performed well as a thermal insulating material with a conductivity between 0.0642 and 0.0676 W/m·K and a classification of Bd0 according to the European standards on fire resistance; some of them may be used to manufacture furniture, interior décor, and load-bearing panels in dry conditions.

## 1. Introduction

Due to the deforestation that is occurring, we need to seek suitable replacements for wood, for use in both cellulose derivatives and building elements. The causes of this loss of forests include the expansion of pastures and crops, urban development, and wood production for industrial uses and as fuel.

From the technical point of view, non-woody plants’ fibers offer a large variety of qualities and, if adequately exploited, they can be used to develop materials with innovative properties to replace wood.

Agriculture generates waste that is not currently managed properly in terms of environmental and economic aspects. Apart from reducing and recycling agricultural waste, co-products, and by-products, there may be opportunities for new processes that result in innovative uses of such waste. According to the International Organisation of Vine and Wine (OIV) [1], 7.6 million hectares of the world’s surface were covered by vineyards in 2017, of which approximately 1 million hectares are in Spain.

It is important to remove vine waste from the fields in order to prevent the spread of fires, pests, or diseases. It is common practice to dispose of this waste by open burning, which has a significant environmental impact in the form of greenhouse gas (GHG) emissions, other pollutants, and suspended particles.

Wood and other plant materials have been used in traditional buildings for their strength and insulating properties, and replacements are currently being sought in response to this scarcity of wood. Therefore, using plant biomass in buildings will contribute to greater energy efficiency and less environmental pollution.

The greatest challenge when working with plant fibers is the considerable variation in their thermal properties and characteristics, which depend on their complex structural geometric architectures [2]. Numerous studies have been carried out on the insulating properties of plant waste: Coconut fiber [3], paper manufacturing waste and corn peel [4], kenaf fibers [5], cotton stalk fibers [6,7], coconut husk and bagasse [8], hemp fibers [9,10], date palm fibers and gypsum [11], flax [12], flax and hemp [13], rice straw [14], sisal [15], sugarcane bagasse [16], giant reed [17,18,19], Canary Islands palms [20], and Washingtonia palms [21].

Research has been carried out on the use of vine prunings to manufacture particleboards using urea-formaldehyde (UF) resin as an adhesive [22]. Vine pruning particles have been subjected to a chemical pretreatment and the proportions of UF used as a binder [23] evaluated in terms of the mechanical properties achieved by the particleboards. Vine pruning particles have been mixed with pine particles and boards made using UF, obtaining poor bending strength and high thickness swelling values [24]. Boards made from pine wood and several different proportions of vine prunings have also been evaluated, concluding that small amounts of these particles could be included to manufacture boards for furniture production [25].

An important issue with building materials made from plant fibers is the lack of information about their reaction to fire. Renewable building materials have the potential to partially replace commonly used materials such as cement, but they must meet important requirements. Fire safety must be addressed in accordance with the Construction Products Regulation (EU) No. 305/2011 (CPR) [26]. Some studies have been carried out to investigate the reaction to fire of particleboards made from or containing agricultural waste such as flax [27], oil palm [28], kenaf [29], and rice straw [14], but no information is available in the literature about how vine particleboards react to fire.

The cited works that use vine prunings to manufacture particleboards focus on analyzing the mechanical and physical properties, but we did not find any references that explore their thermal or fire-resistance properties. The objective of this work was to obtain a product by using vine pruning waste to make particleboards and assess their use as an insulating material and as a fire-resistant material.

## 2. Materials and Methods

The materials used were shredded and sieved vine shoot pruning waste that came from the Higher Technical College of Orihuela at Universidad Miguel Hernández in Elche, Spain. The pruning waste was left to dry outdoors for six months. It was then shredded in a blade mill. The particles were collected in a vibrating sieve and two particle sizes were selected: Particles that passed through the 2-mm sieve but were retained in the 1-mm one (1 to 2 mm) and particles that passed through the 1-mm sieve but were retained in the 0.25-mm one (0.25 to 1 mm). The approximate moisture content of the particles was 8%. The binder used was 9% by weight (based on the weight of the particles) class E1 urea-formaldehyde (UF) at a concentration of 65% with a reaction time of 3–4 h. Ammonium nitrate was used as a hardener, at a concentration of 0.4% by weight (based on the weight of the particles). No paraffin or water-repellent substance was used. The percentage of UF used was in line with that used in other studies with this material [22,23,24,25]. Eight types of boards (Table 1) were manufactured with vine prunings, using two particle sizes two times and applying two pressures during pressing. The UF resin was sprayed through nozzles onto the particles in an Imal glue blender (model LGB100, Modena, Italy) for 5 min.

The manufacturing process consisted of forming the mat of the board using the vine waste with two different particle sizes. The mat was formed in a mould of dimensions 600 mm × 400 mm and was subjected to pressure and heat in a hotplate press, applying two pressures of 2 and 2.6 MPa at a temperature of 140 °C for 4 and 6 min. The pressing cycle was controlled in terms of pressure. The panels were then left to cool in a vertical position. The production characteristics of the eight types of panels are shown in Table 1. Four panels of each type were manufactured.

The particleboards consisted of a single layer and their approximate dimensions were 600 × 400 × 7.5 mm^3^. Experimental tests were conducted to determine physical, mechanical, thermal, and fire-resistance properties.

**Figure 1:**
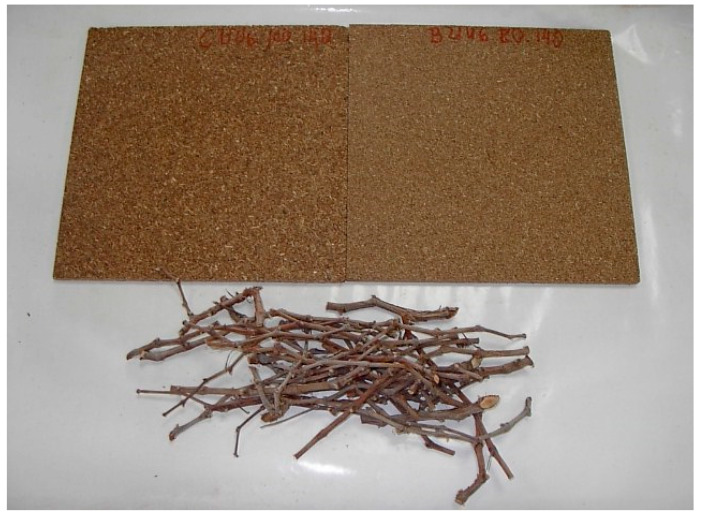
Vine prunings and samples of the manufactured boards (300 × 300 mm^2^).

Figure 1 shows the vine pruning waste used and some samples (300 × 300 mm^2^) of the manufactured boards.

Before testing, the samples were placed in a JP Selecta refrigerated cabinet (model Medilow-L, Barcelona, Spain) for 24 h at a temperature of 20 °C and relative humidity of 65%.

The moisture content was measured with an Imal laboratory moisture meter (Model UM2000, Modena, Italy). The water immersion test was carried out in a heated tank at a water temperature of 20 °C.

Certain physical and mechanical properties were measured in accordance with the appropriate European standards: Density [30], thickness swelling (TS) [31], and water absorption (WA) after 2 and 24 h immersed in water (the procedure used for the WA test was the same as that used in the TS test). Modulus of rupture (MOR) or bending strength, modulus of elasticity (MOE) [32], and internal bonding strength (IB) [33] were measured.

Additionally, thermal conductivity [34] and reaction to fire using a single-flame source [35] were measured. The mechanical tests were performed with the Imal universal testing machine (model IB600, Modena, Italy). The thermal conductivity tests were conducted with a heat flow measuring instrument (NETZSCH Instruments Inc., Burlington, MA, USA). The reaction-to-fire tests were carried out using a flammability meter (CEAST model 1653, Turin, Italy). The particleboards were classified according to the applicable European regulations [36].

The standard deviation was obtained for the mean values of the tests, and analysis of variance (ANOVA) was performed for a significance level α < 0.05. The statistical analyses were performed using SPSS v.26.0 (IBM, Chicago, IL, USA) software from IBM.

## 3. Results and Discussion

### 3.1. Physical Properties

The density, thickness swelling, water absorption, thermal conductivity and thermal resistance results of the boards manufactured are shown in Table 2.

#### 3.1.1. Density

Vine pruning panels were successfully manufactured with densities ranging from 782.94 to 965.06 kg/m^3^; they could, therefore, be classified as boards with a medium-high apparent density. As can be seen in the ANOVA of Table 3, this property depended on particle size and the pressure applied, but the pressing time had no influence.

#### 3.1.2. Thickness Swelling and Water Absorption

Particleboards must have a maximum thickness swelling value of 17% after 24 h immersed in water to be classed as Grade P3 according to the European regulations [37], and there is no minimum TS value in the standards for general use and furniture manufacture in dry conditions (Grades P1 and P2, respectively). 

The TS results showed similar values after 24 h for all board types, ranging from 26.0% to 31.6%. For WA after 24 h, the values ranged from 60.8% to 82.2%. According to the ANOVA performed (Table 3), these properties depended on the particle size and were not influenced by the pressure applied or the pressing time and their values showed us that these boards did not perform well when they were exposed to humid conditions.

In general, the thickness swelling of the boards was very high. This could be both due to lack of water-repellent chemicals in the mat mixtures and due to the large amount of pith [24,25]. The high mean thickness swelling observed for dense particleboards could be explained by the higher number of water attractive OH groups in the material [38].

Thickness swelling over 24 h was greater than that required by the regulations [37] for Grade P3 (17%) in all the experimental panels manufactured in this study. As can be seen in Table 4, the mean TS and WA values obtained in this work were similar to those obtained in other studies using plant fibers.

The high values obtained for WA were due to the high porosity of the board and to not using water-repellent chemicals during the panel’s manufacture. The results achieved were similar to those obtained in another study with vine prunings [22], and it should be stressed that the boards in this work offered better WA properties than those achieved with other plant fibers (Table 4). Moreover, they were obtained at a lower temperature (140 °C) and with a smaller amount of UF (9%).

#### 3.1.3. Thermal Resistance and Conductivity

The results of the thermal conductivity and resistance tests are shown in Table 2, offering similar values for all board types. The thermal conductivity of the experimental panels was between 0.0642 and 0.0676 W/m·K. As shown by the ANOVA in Table 3, these values depended on the particle size and were not influenced by the pressure applied or the pressing time. The larger the particle size, the lower the thermal conductivity obtained, as the porosity of the boards increased.

Table 5 compares the thermal conductivity values obtained by other authors with other plant fibers. In tests with boards of similar densities to those of our study, similar values were obtained.

The natural materials that are used commercially (flax, hemp, cotton, etc.) and soft wood fiber boards (low-density wet-process fiberboard) have better thermal properties than the boards obtained in this work, but these materials are only pressed and they do not have any mechanical strength, so they are used as a filler or coated with other stronger materials. The results obtained in this work were lower than those of wood particleboards. Therefore, they can be considered good thermal insulating panels.

### 3.2. Mechanical Properties

Mechanical strength is not generally one of the main requirements for thermal insulating materials, but in this work the manufacturing parameters were chosen to ensure not only the good thermal performance of the boards obtained, but also that they can be used for applications that require certain mechanical properties in buildings, such as enclosures (vertical and horizontal).

According to the European standards [37], the minimum requirements for general use in dry condition panels are an MOR value of 10.5 N/mm^2^ and an IB value of 0.28 N/mm^2^ (Grade P1). An MOR value of 11 N/mm^2^, an MOE value of 1800 N/mm^2,^, and an IB value of 0.40 N/mm^2^ are the minimum requirements for furniture manufacturing (Grade P2). For load-bearing boards (Grade P3), the MOR, MOE, and IB values are 15 N/mm^2^, 2050 N/mm^2^, and 0.45 N/mm^2^, respectively.

The results of the mechanical tests are shown in Table 6. The MOR values ranged between 6.58 and 16.5 N/mm^2^. The MOE values were between 743 and 1810 N/mm^2^. The results of the IB test ranged from 1.22 to 1.92 N/mm^2^, showing excellent properties in all cases for this parameter, since the minimum required for Grade P7 (heavy-duty load-bearing boards for use in humid conditions) is 0.75 N/mm^2^, although the other conditions required by the regulations were not met [37]. The mechanical properties were highly dependent on the particle size and the pressure applied. The mechanical values for MOE and MOR increased when the pressure and pressing time were increased, but pressing time did not influence these values. The IB also depended on the pressing time. The boards with the best mechanical performance were type 3 and 4 boards, with a particle size of 0.25 to 1 mm and 2.6 MPa of pressure, after 4 and 6 min of pressing, respectively.

All boards with a particle size of 0.25 to 1 mm could be classed as Grade P1, for general use in dry conditions. The only board that could be classed as Grade P2 (interior fitments including furniture in dry conditions) would be type 4.

The results showed that the MOR and MOE values improved when the particle size was smaller. These results are in line with those obtained by other authors [47]. 

The values achieved in this work for IB were better than those found in other studies [23,24,25], obtaining the best result using a smaller particle size and a greater pressure and pressing time. This shows that it is possible to produce boards for industrial purposes with a process that uses a low temperature and a small percentage of UF.

### 3.3. Reaction-to-Fire Test Results

**Figure 2:**
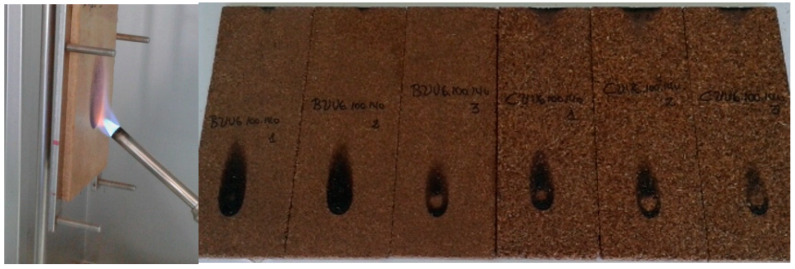
Sample placed on the frame and samples used in the reaction-to-fire test.

Three samples of each type of board were used to carry out the reaction-to fire-test. The samples were prepared before the test at a constant mass, a temperature of (23 ± 2 °C) and a relative humidity of (60% ± 5%). Figure 2 shows the flame being applied to a sample and some of the samples tested.

The results are shown in Table 7. Flame spread, Fs, is a measure of flame height. As can be seen in Figure 2, the burned area was superficial. The result was similar for all the boards tested. The regulations establish that when Fs < 150 mm in 60 s, boards are classified as B. Products classed as B have an additional classification, s2, if they do not produce smoke. If, additionally, there were no flaming droplets, as was the case, the boards are classified as d0. The vine pruning particleboards were, therefore, classed as B-s2 d0.

Flammability tests should be performed to determine whether the boards could be classified in a higher class. Wood particleboards without additives are class Dd0, which means that their reaction to fire is worse than that of the vine shoot particleboards manufactured in this work. This could be explained by the silica content in the material, as silica is known to be a fire retardant [28,29], although to confirm this it would be necessary to perform an analysis of sand content [48] of the boards manufactured.

There are seven fire classes according to the applicable European regulations [36]. Classification is a means of considering the extent to which the building material contributes to the generation and spread of fire and smoke within the room of origin or in a given area. Products are generally considered in relation to their end-use application. Class A is for products that will not contribute to the fire load and growth. Class F is for products for which no reaction-to fire-performances are determined or which cannot be classified in one of the other classes. Class B is like Class C, but satisfying more stringent requirements. According to their end use, wood-based panels are Class D, but based on the results of the test, the experimental panels should be classified as Class B, the same category as gypsum boards, fire-retardant wood, and fire-retardant polymers.

## 4. Conclusions

The results show that it is possible to produce particleboards using vine pruning waste as a raw material.

The thickness swelling and water absorption values were relatively high, so adding water-repellent chemicals during manufacture of the board would significantly improve these parameters.

All the manufactured boards met the minimum requirements for medium-density particleboards and they also had good thermal insulation properties, with an average conductivity of 0.066 W/m·K.

Higher pressure and smaller particle size resulted in better mechanical properties. All panels with a particle size of 0.25 to 1 mm could be classed as Grade P1 for general use in dry conditions. The type 4 board could be classed as Grade P2 for the manufacture of furniture, interior décor, and enclosures (vertical and horizontal) in dry conditions.

The reaction-to-fire test showed that vine pruning particleboards can be considered fire retardant, offering better performance than wood boards.

The use of this waste to manufacture long-lasting products not only contributes to the development of more sustainable materials, but also has great environmental benefits.

## Figures and Tables

**Table 1:** Types of board manufactured.

Type of Board	Particle Size (mm)	Pressing Pressure (MPa)	Pressing Time (min)
1	0.25 to 1	2.0	4
2	0.25 to 1	2.0	6
3	0.25 to 1	2.6	4
4	0.25 to 1	2.6	6
5	1 to 2	2.0	4
6	1 to 2	2.0	6
7	1 to 2	2.6	4
8	1 to 2	2.6	6

**Table 2:** Average results of physical and thermal properties.

Type of Board	Thickness (mm)	Density (kg/m^3^)	TS 2 h (%)	TS 24 h (%)	WA 2 h (%)	WA 24 h (%)	Thermal Conductivity (W/(m·K))	Thermal Resistance (m^2^·K/W)
1	7.46 (0.55)	865.36 (27.81)	15.4 (1.8)	26.9 (3.3)	38.8 (10.8)	60.8 (10.6)	0.0673 (0.0005)	0.1041 (0.008)
2	7.25 (0.74)	850.46 (18.59)	15.7 (2.3)	26.0 (2.1)	48.9 (12.2)	77.0 (9.1)	0.0645 (0.0009)	0.1086 (0.015)
3	7.55 (0.90)	911.69 (44.75)	17.2 (1.0)	29.6 (3.2)	38.8 (11.0)	71.5 (6.6)	0.0646 (0.0011)	0.1086 (0.015)
4	7.49 (0.39)	965.06 (41.36)	16.4 (3.9)	27.2 (2.7)	37.3 (8.0)	64.3 (7.2)	0.0676 (0.0020)	0.1036 (0.031)
5	7.45 (0.22)	782.94 (34.64)	22.0 (3.3)	30.4 (0.4)	52.8 (26.59)	79.1 (13.3)	0.0642 (0.0005)	0.1090 (0.009)
6	7.48 (0.37)	793.32 (37.11)	21.3 (1.5)	29.0 (3.4)	52.5 (11.3)	82.2 (7.3)	0.0648 (0.0007)	0.1079 (0.013)
7	7.53 (0.70)	820.87 (41.65)	18.8 (8.7)	31.6 (2.3)	36.6 (6.1)	77.1 (3.8)	0.0654 (0.0001)	0.1079 (0.013)
8	7.53 (0.29)	846.89 (30.58)	21.8 (3.8)	30.2 (1.9)	44.1 (10.7)	75.6 (6.0)	0.0647 (0.0006)	0.1083 (0.009)

TS: Thickness swelling. WA: Water absorption. (.): Standard deviation.

**Table 3:** ANOVA of the results of the tests.

Factor	Properties	Sum of Squares	d.f.	Half Quadratic	F	*p*-Value
Particle size	Density (kg/m^3^)	69,936.105	1	69,936.105	27.262	0.000
	TS 24 h (%)	74.295	1	74.295	10.442	0.003
	WA 24 h (%)	1,253.409	1	1,253.409	15.336	0.000
	MOR (N/mm^2^)	349.256	1	349.256	109.665	0.000
	MOE (N/mm^2^)	3,393,780.874	1	3,393,780.874	91.376	0.000
	IB (N/mm^2^)	0.765	1	0.765	13.975	0.001
	Thermal C. (W/m·K)	0.0000155	1	0.0000155	8.882	0.009
	Thermal R. (m^2^·K/m)	0.0000249	1	0.0000249	4.786	0.044
Pressing pressure	Density (kg/m^3^)	40,607.929	1	40,607.929	11.932	0.001
	TS 24 h (%)	18.187	1	18.187	2.086	0.158
	WA 24 h (%)	56.143	1	56.143	0.484	0.491
	MOR (N/mm^2^)	55.064	1	55.064	4.747	0.036
	MOE (N/mm^2^)	894,968.820	1	894,968.820	6.891	0.013
	IB (N/mm^2^)	0.280	1	0.280	4.382	0.049
	Thermal C. (W/m·K)	0.0000010	1	0.0000010	0.417	0.527
	Thermal R. (m^2^·K/m)	0.0000013	1	0.0000013	0.194	0.666
Pressing time	Density (kg/m^3^)	2,258.231	1	2,258.231	0.502	0.483
	TS 24 h (%)	5.773	1	5.773	0.636	0.430
	WA 24 h (%)	196.307	1	196.307	1.754	0.194
	MOR (N/mm^2^)	11.194	1	11.194	0.871	0.357
	MOE (N/mm^2^)	105,484.796	1	105,484.796	0.692	0.411
	IB (N/mm^2^)	0.280	1	0.280	4.091	0.048
	Thermal C. (W/m·K)	0.0000001	1	0.0000001	0.052	0.822
	Thermal R. (m^2^·K/m)	0.0000012	1	0.0000012	0.194	0.666

d.f.: Degrees of freedom. F: Fisher–Snedecor distribution.

**Table 4:** Thickness swelling (TS) values obtained with plant fiber boards.

Name	TS 24 h (%)	WA 24 h (%)	Source
Tobacco straw	22.0	-	[39]
Cotton stalks	24.0	93.6	[40]
Sunflower stalk	25.0	95.0	[41]
Cotton carpel	26.0	153	[42]
Wheatgrass	41.7	-	[43]
Vine prunings	25.8	65.6	[22]
	28.9	73.4	Mean values in this work

**Table 5:** Thermal conductivity coefficients obtained in tests with different organic fibers.

Name	Thermal Conductivity λ (W/m K)	Source
Hemp	0.111	[9]
	0.040 to 0.094	[13]
Flax	0.038 to 0.075	[13]
	0.042	[3]
Cotton	0.040 to 0.069	[44]
Date palm rachis	0.083	[45]
Rice straw	0.076 to 0.091	[14]
Sisal	0.070	[15]
Sugarcane bagasse	0.079 to 0.098	[16]
Wood particleboards	0.070 to 0.180	[46]
Wood fiberboards	0.050 to 0.140	[46]
Vine prunings	0.064 to 0.068	This work

**Table 6:** Mean values of mechanical properties.

Type of Board	MOR (N/mm^2^)	MOE (N/mm^2^)	IB (N/mm^2^)
1	13.6 (0.4)	1,390 (87)	1.32 (0.09)
2	12.4 (1.0)	1,300 (201)	1.22 (0.05)
3	15.3 (0.5)	1,800 (36)	1.70 (0.14)
4	16.5 (0.8)	1,810 (58)	1.79 (0.13)
5	8.72 (0.6)	963 (106)	1.92 (0.15)
6	6.58 (1.0)	743 (59)	1.86 (0.08)
7	8.88 (0.6)	1,030 (73)	1.45 (0.04)
8	9.22 (0.6)	1,080 (62)	1.84 (0.14)

MOR: Modulus of rupture. MOE: Modulus of elasticity. IB: Internal bonding strength. (.): Standard deviation.

**Table 7:** Mean flame spread (Fs) results with respect to the type of board.

Type of Board	1	2	3	4	5	6	7	8
**Weight loss (%)**	0.28 (0.04)	0.17 (0.02)	0.14 (0.01)	0.18 (0.03)	0.15 (0.04)	0.22 (0.02)	0.12 (0.01)	0.15 (0.03)
**Burn height (mm) (Fs)**	67.78 (1.17)	48.96 (2.43)	57.45 (1.75)	45.60 (1.68)	46.60 (0.94)	47.28 (0.71)	43.65 (1.75)	41.04 (0.51)
**Burn width (mm)**	23.12 (0.44)	20.38 (0.67)	22.25 (0.19)	20.70 (0.82)	19.81 (1.61)	21.28 (0.69)	20.37 (0.35)	20.01 (0.15)
**Board ignition**	No	No	No	No	No	No	No	No
**Filter paper ignition**	No	No	No	No	No	No	No	No
**Smoke**	No	No	No	No	No	No	No	No

(.): Standard deviation.
